# Effects of Yoga and Walking on Blood Glucose Levels and Quality of Life in Adults With Type 2 Diabetes Mellitus: A Pilot Study

**DOI:** 10.7759/cureus.97023

**Published:** 2025-11-16

**Authors:** Darshana Hazarika, Imran Khan, Mangala Lahkar

**Affiliations:** 1 Research, Sharda School of Nursing Science and Research, Sharda University, Greater Noida, IND; 2 NEMCARE Institute of Nursing Sciences, NEMCARE Group of Institutions, Guwahati, IND; 3 Nursing, Sharda School of Nursing Science and Research, Sharda University, Greater Noida, IND; 4 Pharmacology, Srimanta Sankaradeva University of Health Sciences, Guwahati, IND

**Keywords:** adults, quality of life, type 2 diabetes mellitus, walking, yoga

## Abstract

Background

Type 2 diabetes mellitus (T2DM) is a chronic metabolic disease primarily caused by a combination of two main factors: defective insulin secretion by pancreatic β-cells and the inability of insulin-sensitive tissues to respond to insulin. Yoga and walking are effective strategies for the management of T2DM and the promotion of wellness in diabetics.

Materials and methods

A quasi-experimental design was adopted to evaluate the effect of yoga and walking on blood glucose levels and quality of life in adults with T2DM. Data were collected from December 2, 2023, to March 3, 2024, from the Mirza areas of the Kamrup Rural district of Assam. This pilot study included 20 persons with type 2 diabetes who were divided into four groups of five each. Group I did yoga as an intervention, Group II did walking as an intervention, and Group III combined yoga and walking on alternate days as an intervention. Group IV was the control group. A multistage sampling technique was adopted to select samples that met specific inclusion criteria.

Results

There was a significant improvement in fasting blood sugar (FBS) levels for the yoga (F = 9.48, p = 0.002) and walking (F = 8.68, p = 0.002) groups. However, the postprandial blood sugar (PPBS) score was only significant in the yoga group (F = 35.34, p = 0.004). Over time, all three intervention groups had a statistically significant drop in HbA1c levels (p-values = 0.03, 0.03, and 0.02, respectively). Regarding quality-of-life scores, the differences between group categories were not statistically significant (p > 0.05). Concurrently with the group comparison, domains were also compared: in the psychological domain, the walking group exhibited a highly significant improvement (F = 28.89, p < 0.006). The walking group was the only one to demonstrate highly significant development in the social relationship domain (F = 28.89, p = 0.006). The yoga groups exhibited significant improvements in the environmental domain (F = 125.88, p = 0.001).

Conclusion

Across all intervention groups, the results suggest a substantial time-dependent decrease in acute blood glucose levels (FBS and PPBS), with the combined yoga and walking group exhibiting the most substantial improvements.

## Introduction

Type 2 diabetes mellitus (T2DM) is a chronic metabolic disease primarily caused by two main factors: defective insulin secretion by pancreatic β-cells and the inability of insulin-sensitive tissues to respond to insulin [1]. Globally, T2DM, which is frequently characterized by decreased insulin production and insulin resistance, negatively impacts patients’ quality of life (QoL). Patients with T2DM experience numerous potentially fatal health complications, which increase medical costs, decrease QoL, and raise mortality risk [2]. Obesity is a pervasive global issue that is currently a problem in every nation, and it is expected to become even more significant over the next decade, resulting in a greater loss of healthy life, disability, and death. To prevent and treat obesity and related comorbidities, such as T2DM, which is a medical condition that is spreading at an alarming rate worldwide, it is imperative to implement effective and swift measures to curb its rising prevalence [3]. The treatment of T2DM requires medication, a balanced diet, and physical activity. Well-organized, evidence-based, and supervised physical exercise is an affordable therapeutic approach for managing T2DM [4]. The best choices to control diabetes in underdeveloped nations are to use low-cost lifestyle interventions like yoga and low-cost measures to identify at-risk individuals. Regular exercise, particularly yoga, appears to be a highly beneficial and affordable adjuvant therapy for T2DM [5]. The American Diabetes Association recognizes yoga as a beneficial physical activity for patients with diabetes. Yoga is an ancient discipline that aims to balance and harmonize the body, mind, and emotions [6]. Yoga therapy is more effective in promoting balance, reducing fatigue, and increasing interaction between individuals and society. Yoga and walking are effective strategies for the management of T2DM and the promotion of wellness in diabetics [7]. People with type 2 diabetes who practiced yoga and went for walks saw an improvement in their clinical and laboratory indicators [8].

Numerous trials have demonstrated that yoga improves outcomes in diabetes. Some studies have reported benefits exceeding 50% for yoga interventions in managing diabetes [9]. It has been practiced for a long time, but using it as therapy is still a new trend in the healthcare profession. Yoga is a science of health management, not a therapy for treating specific conditions. It is a mind-body practice with the ultimate objective of spiritual enlightenment [10,11].

Walking is the most ancient form of exercise, and it is simple to incorporate into daily life. For many people, it might be the first basic step toward changing their lifestyle. Recent research of postmenopausal women who were at high risk for T2DM showed that both standing and walking quickly lowered blood sugar after eating [12,13].

QoL is a way to quantify how someone evaluates their life. This perspective makes it clear that the most important part of judging QoL is recording the way the person feels about their QoL, not what other people think it should be [14].

Diabetes impacts QoL by elevating the risks of nephropathy, eyesight impairment, cardiovascular issues, and financial strain. The Quality of Life in Diabetic Patients Scale evaluates eight domains of QoL for Indian diabetic patients: physical health, endurance, general health, treatment satisfaction, symptom-specific concerns, financial impact, mental-emotional health, and diet adherence [15].

A study investigated the impact of a pedometer-based walking intervention, conducted thrice weekly for 12 weeks, on clinical, diabetes-related cognitive and social aspects, as well as QoL outcomes in individuals with T2DM. The results indicated that walking positively influenced the patients’ physical activity, HbA1c levels, social incentives for self-care activities, diabetes-related self-efficacy, outcome expectancies, general health perceptions, mental health, and well-being [16].

Yoga has some health benefits, such as improving blood circulation, relieving stress, reducing blood sugar levels (BSLs), reducing weight, and improving overall QoL. Walking for 30 to 60 minutes three or four times a week can help diabetics lower their BSLs. Numerous studies that examined the effects of combined yoga and walking groups produced a variety of conclusions. This study aims to establish clear evidence of the impact of yoga and walking on levels of blood glucose as well as QoL among individuals with T2DM.

## Materials and methods

Materials and methods

Research Design

A quasi-experimental design was adopted to evaluate the effect of yoga and walking on blood glucose levels and QoL among adults with T2DM.

Ethical Considerations

Ethical approval was obtained from the Institutional Ethics Committee (NGI/Ethics/PhD/2023/001), NEMCARE Group of Institutions, Mirza, on July 12, 2023. Administrative permission was obtained from the joint director of Kamrup, Rural Assam. This study was registered under the Clinical Trial Registry of India. Trial No.: CTRI/2023/12/060729.

Study Setting, Sample Size, and Sampling Technique

**Figure 1:**
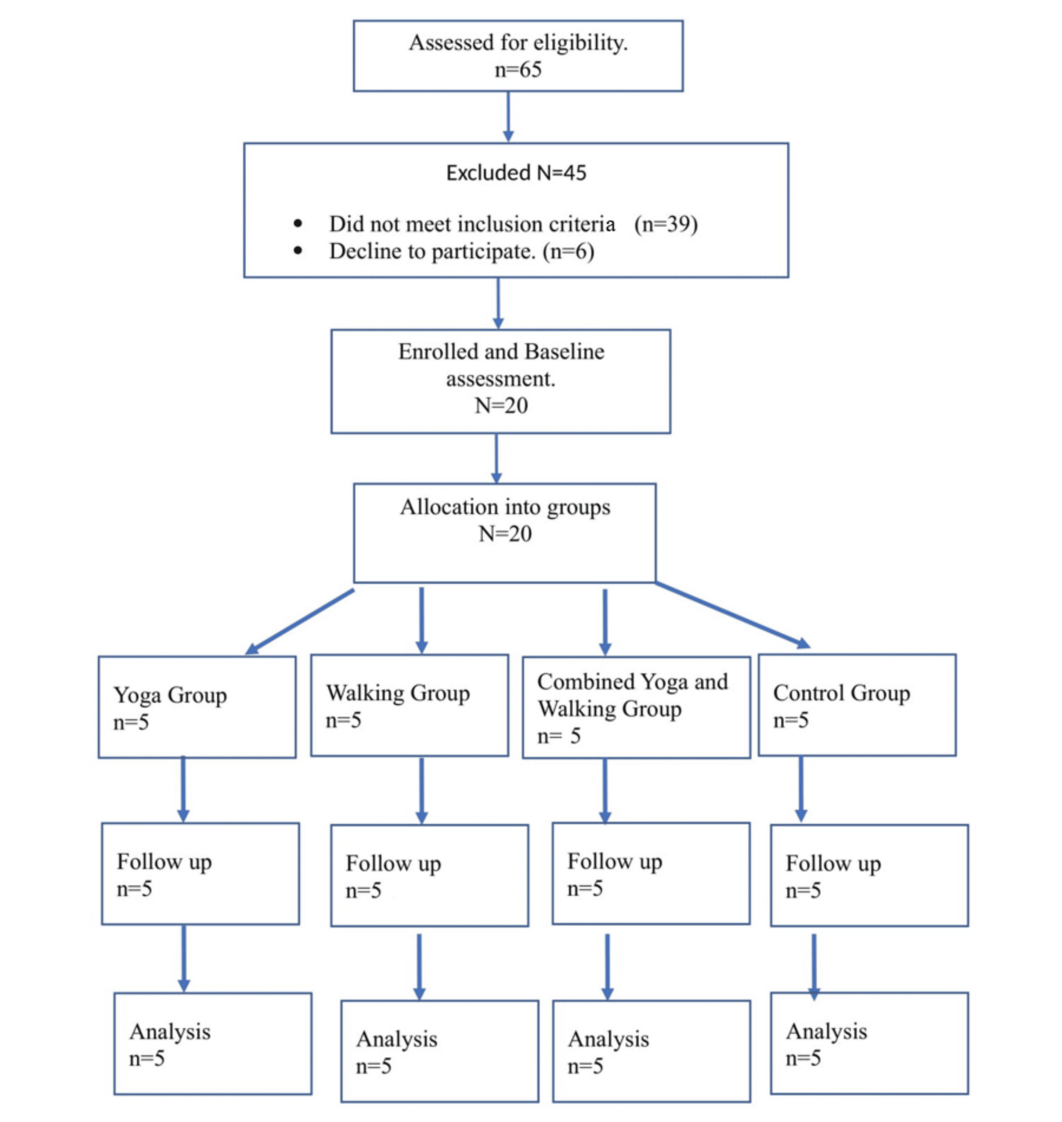
CONSORT diagram of the sample selection CONSORT: Consolidated Standards of Reporting Trials

Data were collected from December 2, 2023, to March 3, 2024, in the Mirza areas of the Kamrup Rural district of Assam. A sample size of 200 was chosen for the main study. To ensure the feasibility and validity of the research design, 20 persons with type 2 diabetes were recruited, constituting 10% of the overall sample size. They were divided into four groups of five each. Group I did yoga as an intervention, group II did walking as an intervention, and group III did combined yoga and walking as an intervention. Group IV was the control group. A multistage sampling technique was adopted to select samples that met specific inclusion criteria. This study enlisted participants who consented to participate, were 18 years of age, and had a confirmed diagnosis of type 2 diabetes. This study excluded participants with a history of mental illness, psychiatric problems, or any other major health problems. Each participant received comprehensive information regarding the research protocol’s objectives and the methodology to be employed. The study flow diagram, according to the Consolidated Standards of Reporting Trials (CONSORT) guidelines [17], is presented in Figure 1.

Inclusion criteria

The inclusion criteria for this study comprised adult patients aged 30-60 years who were previously diagnosed with T2DM and were actively undergoing treatment with oral hypoglycemic agents. Biochemical confirmation was mandated, requiring a fasting blood glucose ≥ 126 mg/dL, a postprandial blood glucose ≥ 200 mg/dL, or an HbA1c level ≥ 6.5%. Additionally, only those individuals who provided informed consent to participate and demonstrated proficiency in understanding and speaking Assamese, Hindi, or English were enrolled.

Exclusion criteria

The exclusion criteria were established to isolate the study population from significant confounding variables. Individuals were deemed ineligible if they had comorbid systemic conditions, including chronic kidney disease, cardiovascular diseases, or active cancer. Furthermore, exclusion extended to those currently participating in other diabetes intervention programs, those prescribed insulin therapy, and any individuals who would not be accessible for the duration of the data collection phase.

Intervention

Yoga Therapy Group

The yoga group respondents were trained to practice yoga intervention from 6 a.m. to 7 a.m. for 60 minutes five times a week for 12 weeks under the supervision of a researcher. Yogasanas included in this study were warm-up, Mandukasana, Shashankasana, Gomukhasana, Ardha Matsyendrasana, Vakrasana, Pawanmuktasana, Dhanurasana, Anulom Vilom Pranayama, Kapalbhati Pranayama, and Bhastrika Pranayama.

Walking Therapy Group

Respondents in the walking therapy group were instructed to practice walking under the supervision of a researcher from 6 a.m. to 7 a.m. for 30 minutes five times a week for 12 weeks.

Combined Yoga and Walking Therapy Alternate Day Group

Respondents in this group were instructed to practice yoga for 60 minutes and walk for 30 minutes on alternate days from 6 a.m. to 7 a.m. under the supervision of a researcher five times a week for 12 weeks.

Control Group

Participants in the control group were instructed to maintain their usual routine care.

To control for confounding variables, all four groups received the same standardized dietary advice at baseline and were instructed to maintain their existing medication regimens, which were monitored throughout the study period.

Assessment of the Outcomes

The primary outcome measure was the blood glucose levels (fasting blood sugar (FBS), postprandial blood sugar (PPBS), and HbA1c) and the QoL of persons with type 2 diabetes. At first, the researcher gathered demographic data from the samples using a self-structured demographic questionnaire. The researcher personally measured the FBS and PPBS levels using a digital glucometer, whereas the HbA1c level was evaluated in a laboratory setting. The World Health Organization Quality of Life Questionnaire (WHOQOL-BREF) was used to gather information about QoL. It consists of 26 questions that are separated into four categories: physical, psychological, environmental, and social. The scores were converted on a scale of 0 to 100. A better QoL is indicated by a higher score [18]. Permission to use the WHOQOL instruments was obtained. The posttest was carried out on BSLs and QoL after the fourth, eighth, and 12th weeks of treatment.

Statistical Analysis

The analysis of the data was done based on the study’s goals. Analyses were performed by using the Statistical Package for the Social Sciences (SPSS) software version 20.0 (IBM Corp., Armonk, NY, US). Descriptive and inferential statistics were employed. We used the frequency and percentage distribution for all the groupings to examine the demographic data. We utilized the means and standard deviations to compare the BSLs and QoL scores between the groups. To assess the homogeneity of the outcome variables (blood glucose level and QoL) at baseline, a one-way ANOVA was conducted. To statistically evaluate the efficacy of interventions on outcome variables at various time points, repeated measures ANOVA was employed. The level of statistical significance was set at p < 0.05.

## Results

The demographic and baseline characteristics of the study participants are summarized in Table 1. The majority of participants across all groups (yoga, walking, combined yoga and walking, and control) were aged 31-40 years. Women comprised the majority in all groups, with the highest proportion (100%) in the control group. Most participants were married, with the yoga and combined groups showing 100% marriage rates. Occupational status was predominantly “clerical/shop/farm” for all groups. A majority reported working two hours or less per day. Educational attainment was primarily at the primary level. A confirmed five- to 10-year history of diabetes mellitus was prevalent across groups. All participants reported a sedentary lifestyle and were non-smokers and non-alcoholic. None performed regular physical exercise. The majority reported no family history of diabetes mellitus and followed a non-vegetarian diet.

**Table 1:** Frequency and percentage distribution of baseline sociodemographic variables among type 2 diabetic adults in the yoga group, walking group, combined yoga and walking group, and control group (N = 20) p > 0.05: not significant; NS: not significant; NA: not applicable

Sociodemographic variables	Yoga group (n = 5)	Walking group (n = 5)	Combined yoga and walking group (n = 5)	Control group (n = 5)	Chi square (ꭓ^2^)	p-value
	Frequency (%)	Frequency (%)	Frequency (%)	Frequency (%)		
Age					ꭓ^2^ = 0.65	p = 0.88 (NS)
31–40 years	3 (60)	4 (80)	3 (60)	3 (60)		
>40 years	2 (40)	1 (20)	2 (40)	2 (40)		
Gender					χ^2^ = 4.76	p = 0.19 (NS)
Male	3 (60)	1 (20)	2 (40)	0		
Female	2 (40)	4 (80)	3 (60)	5 (100)		
Transgender	0	0	0	0		
Marital status					χ^2^ = 11.20	p = 0.01
Single	0	3 (60)	0	4 (80)		
Married	5 (100)	2 (40)	5 (100)	1 (20)		
Occupation	0	0	0	0	χ^2^ = 4.31	p = 0.23 (NS)
Unemployed	0	0	0	0		
Unskilled workers	0	0	0	0		
Skilled workers	1 (20)	2 (40)	0	0		
Clerical/shop/farm	4 (80)	3 (60)	5 (100)	5 (100)		
Professional	0	0	0	0		
Hours of work					χ^2^ = 12	p = 0.21 (NS)
≤1 hour	3 (60)	4 (80)	2 (40)	1 (20)		
2–6 hours	1 (20)	1 (20)	0	0		
7–11 hours	0	0	1 (20)	3 (60)		
≥12 hours	1 (20)	0	2 (40)	1 (20)		
Educational qualification					χ^2^ = 7.61	p = 0.26 (NS)
No formal education	2 (40)	0	0	1 (20)		
Primary education	3 (60)	3 (60)	4 (80)	4 (80)		
Middle school	0	2 (40)	1 (20)	0		
Lifestyle					χ^2^ = 2.22	p = 0.52 (NS)
Sedentary	0	1 (20)	1 (20)	0		
Moderate	5 (100)	4 (80)	4 (80)	5 (100)		
Heavy	0	0	0	0		
Dietary pattern					χ^2^ = 2.22	p = 0.52 (NS)
Vegetarian	1 (20)	1 (20)	0	0		
Non-vegetarian	4 (80)	4 (80)	5 (100)	5 (100)		
Family history of diabetes					χ^2^ = 2.93	p = 0.40 (NS)
Yes	2 (40)	0	2 (40)	1 (20)		
No	3 (60)	5 (100)	3 (60)	4 (40)		
Do you smoke?					NA	
Yes	0	0	0	0		
No	5 (100)	5 (100)	5 (100)	5 (100)		
Do you consume alcohol?					NA	
Yes	0	0	0	0		
No	5 (100)	5 (100)	5 (100)	5 (100)		
Do you perform regular physical exercise?					NA	
Yes	0	0	0	0		
No	5 (100)	5 (100)	5 (100)	5 (100)		

The baseline glycemic characteristics for all study groups are presented in Table 2. At the outset of the study, the combined yoga and walking group exhibited the highest mean levels of FBS, PPBS, and HbA1c. The control and yoga groups demonstrated comparatively lower baseline values across all three glycemic measures.

**Table 2:** Comparison of the baseline outcome variables (blood glucose level) among type 2 diabetic adults in the yoga group, walking group, combined yoga and walking group, and control group (N = 20) p > 0.05: not significant; NS: not significant; FBS: fasting blood sugar; PPBS: postprandial blood sugar; SD: standard deviation

Outcome variables	Yoga group (n = 5)	Walking group (n = 5)	Combined yoga and walking group (n = 5)	Control group (n = 5)	One-way ANOVA (F)	p-value
	Mean ± SD	Mean ± SD	Mean ± SD	Mean ± SD		
FBS (mg/dL)	181.6 ± 12.61	229 ± 67.91	266.3 ± 94.42	175.4 ± 44.32	F = 2.22	p = 0.12 (NS)
PPBS (mg/dL)	275.2 ± 30.62	322.8 ± 157.09	334.8 ± 104.23	254.8 ± 46.51	F = 0.75	p = 0.53 (NS)
HbA1c (%)	8.2 ± 1.48	8.8 ± 1.48	9.4 ± 1.67	7.6 ± 1.32	F = 1.33	p = 0.29 (NS)

As presented in Table 3, the mean pretest QoL scores for all domains were uniform across all study groups. The social relationship domain recorded the highest baseline score (76.20 ± 13.7), whereas the environmental domain recorded the lowest (48.80 ± 2.68).

**Table 3:** Comparison of the baseline outcome variables (QoL) among type 2 diabetic adults in the yoga group, walking group, combined yoga and walking group, and control group (N = 20) p > 0.05: not significant; NS: not significant; SD: standard deviation; QoL: quality of life

Outcome variables	Yoga group (n = 5)	Walking group (n = 5)	Combined yoga and walking group (n = 5)	Control group (n = 5)	One-way ANOVA (F)	p-value
Quality of life	Mean ± SD	Mean ± SD	Mean ± SD	Mean ± SD	F = 0.74	p = 0.53 (NS)
Physical	62.8 ± 10.82	62.8 ± 10.82	62.8 ± 10.82	62.8 ± 10.82		
Psychological	61.4 ± 16.07	61.4 ± 16.07	61.4 ± 16.07	61.4 ± 16.07	F = 0.45	p = 0.71 (NS)
Social relationship	76.2 ± 13.71	76.2 ± 13.71	76.2 ± 13.71	76.2 ± 13.71	F = 0.92	p = 0.45 (NS)
Environmental	48.8 ± 2.68	48.8 ± 2.68	53.8 ± 9.49	50.1 ± 7.24	F = 0.53	p = 0.66 (NS)

An analysis of blood glucose levels across three post-intervention assessments is presented in Table 4. All three intervention groups (yoga, walking, and combined) demonstrated a progressive and notable reduction in mean FBS and PPBS scores from posttest-1 to posttest-3. The most substantial improvements were observed in the combined yoga and walking group. In contrast, the control group showed no meaningful change in FBS, PPBS, or HbA1c levels across the three time points, with values remaining stable. HbA1c levels decreased in the yoga and walking groups by the final assessment. Notably, the combined group and control group’s HbA1c remained unchanged across all posttests. These results indicate a positive time-dependent effect of the interventions on acute glycemic measures (FBS and PPBS) compared to the control.

**Table 4:** Mean and standard deviation (SD) of blood glucose levels among the experimental and control groups in the posttest (N = 20) PPBS: postprandial blood sugar; FBS: fasting blood sugar

Outcome variables	Yoga group (n = 5)	Walking group (n = 5)	Combined yoga and walking group (n = 5)	Control (n = 5)
	Mean ± SD	Mean ± SD	Mean ± SD	Mean ± SD
FBS				
Posttest-1	178.4 ± 14.12	226 ± 66.42	256.2 ± 102.22	175.4 ± 44.34
Posttest-2	169.4 ± 24.13	211 ± 60.72	192 ± 70.32	175.8 ± 44.82
Posttest-3	155.8 ± 19.52	176.4 ± 44.12	161.6 ± 43.62	175.8 ± 44.81
PPBS				
Posttest-1	275.2 ± 30.62	322.8 ± 157.12	334 ± 104.14	253 ± 47.15
Posttest-2	274 ± 30.17	320.6 ± 158.14	286.4 ± 45.41	260 ± 59.72
Posttest-3	238.2 ± 26.13	263.8 ± 75.71	254.6 ± 24.34	260 ± 59.71
HbA1c				
Posttest-1	8.3 ± 1.29	8.74 ± 1.47	9.3 ± 1.76	7.56 ± 1.34
Posttest-2	8.3 ± 1.29	8.74 ± 1.47	9.3 ± 1.76	7.56 ± 1.34
Posttest-3	7.42 ± 0.81	7.76 ± 1.21	7.54 ± 0.91	7.56 ± 1.34

The changes in QoL domains across the three post-intervention time points are detailed in Table 5. Key trends were observed: The yoga group demonstrated improvements in the physical and psychological domains at posttest-2, which were maintained through posttest-3. The walking group showed a marked increase in psychological QoL and a progressive improvement in social relationships across all posttests. The combined yoga and walking group exhibited a significant increase in psychological QoL scores at posttest-2, which was also maintained. Notably, the environmental domain remained unchanged across all groups and time points. The control group showed no improvement in any QoL domain over the study period, with scores remaining stable across all three posttests. These results suggest that the interventions had a positive and sustained impact on specific psychological and social aspects of QoL, with no observed effect on the environmental domain.

**Table 5:** Mean and standard deviation (SD) of QoL scores among the experimental and control groups in the posttest (N = 20) QoL: quality of life

Outcome variables	Yoga group (n = 5)	Walking group (n = 5)	Combined yoga and walking group (n = 5)	Control (n = 5)
	Mean ± SD	Mean ± SD	Mean ± SD	Mean ± SD
Physical				
Posttest-1	62.8 ± 10.82	69 ± 0.12	62.6 ± 9.01	61.6 ± 10.21
Posttest-2	67.6 ± 8.17	69 ± 0.12	62.6 ± 9.01	61.6 ± 10.21
Posttest-3	67.6 ± 8.17	69 ± 0.12	62.6 ± 9.01	61.6 ± 10.21
Psychological				
Posttest-1	61.4 ± 16.07	71.4 ± 5.36	66.4 ± 22.21	75 ± 9.27
Posttest-2	65 ± 14.31	80 ± 8.24	77.6 ± 9.52	79 ± 11.42
Posttest-3	65 ± 14.31	80 ± 8.24	77.5 ± 9.52	79 ± 11.42
Social relationship				
Posttest-1	81.4 ± 11.71	75.2 ± 10.82	85 ± 11.41	72.6 ± 13.82
Posttest-2	81.4 ± 11.71	75.2 ± 10.82	85 ± 11.41	72.6 ± 13.82
Posttest-3	81.4 ± 11.71	75.2 ± 10.82	85 ± 11.41	72.6 ± 13.82
Environmental				
Posttest-1	63.8 ± 8.28	61.6 ± 3.13	62.8 ± 4.61	63.8 ± 8.28
Posttest-2	63.8 ± 8.28	61.6 ± 3.13	62.8 ± 4.61	63.8 ± 8.28
Posttest-3	63.8 ± 8.28	61.6 ± 3.13	62.8 ± 4.61	63.8 ± 8.28

The data presented in Table 6 show repeated measures ANOVA on blood glucose levels at different time points among type 2 diabetic adults in the yoga group, walking group, combined yoga and walking group, and control group. It was found that outcome variable scores among the groups like FBS (F = 1.32, p = 0.30), PPBS (F = 0.51, p = 0.68), and HbA1c (F = 0.84, p = 0.48) were not statistically significant. There was a significant improvement in FBS levels for the yoga (F = 9.48, p = 0.002) and walking (F = 8.68, p = 0.002) groups. However, the PPBS score was only significant in the yoga group (F = 35.34, p = 0.004). Over time, all three intervention groups had a statistically significant drop in HbA1c levels (p-values = 0.03, 0.03, and 0.02, respectively).

**Table 6:** Repeated measures ANOVA on outcome variables (blood glucose level) at different time points among type 2 diabetic adults in the yoga group, walking group, combined yoga and walking group, and control group (N = 20) * indicates p < 0.05 (level of significance). FBS: fasting blood sugar; PPBS: postprandial blood sugar

Outcome variables	Group	Within the group F and p-value	Between the group F and p-value
FBS	Yoga (n = 5)	F = 9.48, p = 0.002*	F = 1.32, p = 0.30
	Walking (n = 5)	F = 8.68, p = 0.002*	
	Combined yoga and walking (n = 5)	F = 2.41, p = 0.11	
	Control (n = 5)	F = 2.66, p = 0.17	
PPBS	Yoga (n = 5)	F = 35.34, p = 0.004*	F = 0.51, p = 0.68
	Walking (n = 5)	F = 2.23, p = 0.20	
	Combined yoga and walking (n = 5)	F = 2.13, p = 0.21	
	Control (n = 5)	F = 0.28, p = 0.62	
HbA1c	Yoga (n = 5)	F = 10.40, p = 0.03*	F = 0.84, p = 0.48
	Walking (n = 5)	F = 9.28, p = 0.03*	
	Combined yoga and walking (n = 5)	F = 11.81, p = 0.02*	
	Control (n = 5)	F = 0.21, p = 0.58	

The data presented in Table 7 reveal that the QoL scores between group categories were not statistically significant (p > 0.05): physical (p = 0.51), psychological (p = 0.37), social relationship (p = 0.35), and environmental (p = 0.96). Concurrently with the group comparison, in the psychological domain, the walking group exhibited a highly significant improvement (F = 28.89, p = 0.006). The walking group was the only one to demonstrate a highly significant development in the social relationship domain (F = 28.89, p = 0.006). The yoga group exhibited significant improvements in the environmental domain (F = 125.88, p = 0.001).

**Table 7:** Repeated measures ANOVA on outcome variables (QoL) at different time points among type 2 diabetic adults in the yoga group, walking group, combined yoga and walking group, and control group * indicates p < 0.05 (level of significance). QoL: quality of life

Outcome variables	Group	Within the group F and p-value	Between groups F and p-value
Physical	Yoga (n = 5)	F = 4.57, p = 0.09	F = 0.88, p = 0.51
	Walking (n = 5)	F = 0.49, p = 0.69	
	Combined yoga and walking (n = 5)	F = 0.37, p = 0.57	
	Control (n = 5)	F = 2.15, p = 0.23	
Psychological	Yoga (n = 5)	F = 6.00, p = 0.07	F = 1.10, p = 0.37
	Walking (n = 5)	F = 28.89, p = 0.006*	
	Combined yoga and walking (n = 5)	F = 1.88, p = 0.24	
	Control (n = 5)	F = 8.71, p = 0.06	
Social relationship	Yoga (n = 5)	F = 0.37, p = 0.57	F = 1.15, p = 0.35
	Walking (n = 5)	F = 28.89, p = 0.006*	
	Combined yoga and walking (n = 5)	F = 1.62, p = 0.226	
	Control (n = 5)	F = 2.25, p = 0.22	
Environmental	Yoga (n = 5)	F = 125.88, p = 0.001*	F = 0.09, p = 0.96
	Walking (n = 5)	F = 6.34, p = 0.06	
	Combined yoga and walking (n = 5)	F = 37.03, p = 0.006	
	Control (n = 5)	F = 1.89, p = 0.26	

## Discussion

The current study revealed that the outcome variable scores between the groups for FBS (F = 1.32, p = 0.30), PPBS (F = 0.51, p = 0.68), and HbA1c (F = 0.84, p = 0.48) were not statistically significant (p > 0.05). There was a significant improvement in the FBS level for the yoga (F = 9.48, p = 0.002) and walking (F = 8.68, p = 0.002) groups. However, the PPBS score was only significant in the yoga group (F = 35.34, p = 0.004). Over time, all three intervention groups had a statistically significant drop in HbA1c levels (p-values = 0.03, 0.03, and 0.02, respectively). This finding is comparable with the result reported by Esha et al., in which the intra-group comparison for fasting and postprandial BSL for all three groups was significant (p-value < 0.05) [19].

In the present study, the pretest FBS scores of type 2 diabetic adults in the yoga, walking, combined yoga and walking, and control groups were 181.6 ± 12.6, 229.0 ± 67.9, 266.3 ± 94.4, and 175.4 ± 44.3, respectively. The posttest yoga group achieved a score of 155.8 ± 19.5, whereas the walking group achieved a score of 176.4 ± 44. The combined yoga and walking group had a mean of 161.6 ± 43.6, whereas the control group had a mean of 175.8 ± 44.8.

This result is comparable to the finding reported by Yuniartika et al., in which the average FBS levels in the yoga group were pre (217.00) post (187.72) p (0.001); in the walking group pre (209.89) post (193.83) p (0.001); and in the control group pre (221.50) post (225.17) p (0.067) [20]. The present study revealed that the QoL scores between group categories were not statistically significant (p > 0.05): physical (p = 0.51), psychological (p = 0.37), social relationship (p = 0.35), and environmental (p = 0.96). Concurrently with the group comparison, the walking group exhibited a highly significant improvement in the psychological domain (F = 28.89, p = 0.006) and in the social relationship domain (F = 28.89, p = 0.006), whereas the yoga group exhibited significant improvements in the environmental domain (F = 125.88, p = 0.001).

This finding is in line with the results reported by Ashwini et al. in which a statistically significant improvement in the QoL score was observed after four weeks of training in both groups (p = 0.0001). However, a statistically non-significant change was seen in the QoL score in comparison between groups (p = 0.5765) [21].

Limitations

This study had a small sample size (N = 20), which restricts the statistical power and generalizability of the results. Assessment of long-term outcomes may be inadequate because of the brief 12-week intervention period. The utilization of a quasi-experimental design without randomization elevated the likelihood of selection bias.

## Conclusions

The results of this pilot study indicate that yoga and walking interventions, whether implemented individually or in conjunction, are effective non-pharmacological strategies for enhancing glycemic control and specific aspects of QoL in adults with T2DM. Across all intervention groups, the results suggest a substantial time-dependent decrease in acute blood glucose levels (FBS and PBS), with the combined yoga and walking group exhibiting the most substantial improvements. All three intervention groups also attained a statistically significant decrease in HbA1c, an important indicator for long-term glycemic control. The interventions had a positive effect on the psychological and social relationship domains in terms of QoL. The yoga and combined groups demonstrated substantial improvements in the environmental and psychological dimensions, respectively, whereas the walking group demonstrated highly significant improvements in both these areas. Meanwhile, the control group, which received standard care, did not exhibit significant improvements in any glycemic or QoL indicator during the course of the study. Consequently, the integration of structured yoga, walking, or a combination of the two into diabetes management programs can be a cost-effective and beneficial adjuvant therapy to improve clinical outcomes and the well-being of individuals with T2DM. The necessity of a more extensive investigation to verify these discoveries is substantiated by these findings.
